# Roads do not increase carrion use by a vertebrate scavenging community

**DOI:** 10.1038/s41598-018-34224-x

**Published:** 2018-11-05

**Authors:** Jacob E. Hill, Travis L. DeVault, James C. Beasley, Olin E. Rhodes, Jerrold L. Belant

**Affiliations:** 1 Camp Fire Program in Wildlife Conservation, State University of New York College of Environmental Science and Forestry, Syracuse, NY USA; 2 U.S. Department of Agriculture, Animal and Plant Health Inspection Service, Wildlife Services, National Wildlife Research Center, Sandusky, OH USA; 3 Savannah River Ecology Laboratory, University of Georgia, Aiken, SC USA; 4 Warnell School of Forestry and Natural Resources, University of Georgia, Athens, GA USA; 5 Odum School of Ecology, University of Georgia, Athens, GA USA

## Abstract

Wildlife-vehicle collisions introduce a considerable amount of carrion into the environment, but scavenger use of this resource has not been extensively investigated. Scavengers may use roads for reliable foraging opportunities, but might also use roads for other purposes and encounter carrion opportunistically. We examined scavenging of carrion along linear features by placing 52 rabbit carcasses in each of three treatments in forested habitat during winter (Dec 2016-Mar 2017) in South Carolina, USA: roads, power line clearings (linear feature with fewer carcasses than roads due to lack of road kill), and forest interior. We used motion-activated cameras to compare arrival times and presence of vertebrate scavengers among treatments. There was no difference in proportion of carcasses scavenged or scavenger arrival time across treatments. No species arrived at roads quicker than other treatments. Turkey vultures (*Cathartes aura*) and coyotes (*Canis latrans*) scavenged equally across treatments, whereas gray foxes (*Urocyon cinereoargenteus*) scavenged along roads and power lines, but not in forests. We suggest that scavenger use of carrion near roads at this location during winter relates to factors other than carrion availability. Because some scavengers readily consumed carrion on roads, this resource has the potential to influence the ecology of these species.

## Introduction

Human development is increasingly encroaching into natural areas. Roads are an important means by which this occurs as roads fragment habitat^1^, facilitate transport of pollutants into the environment^2^, and aid the spread of invasive species^3,4^. There are 6.2 million km of roads in the United States alone, and an estimated 20% of the land is impacted by the presence of roads^5,6^. Roads are directly responsible for nearly 10% of large mammal mortality in North America^7^ and each year hundreds of millions of vertebrates die on roads worldwide^8–10^. This mortality can have severe impacts on populations by altering sex ratios^11^, decreasing genetic diversity^12^, and jeopardizing population viability^13–15^.

Although considerable research has assessed the effects of roads on wildlife populations, little attention has been focused on the fate of carcasses resulting from vehicle collisions, which result in substantial additions of carrion to the environment. For example, an estimated 500,000 white-tailed deer (*Odocoileus virginianus*) die annually from collisions in the United States^16^. Assuming an average mass of 45 kg per animal^17^, collisions from this species alone represent 22.5 million kg of carrion introduced into the environment each year, and the total carrion on roads greatly exceeds this amount when vehicle collisions of other species are included. The ecological ramifications of this magnitude of carrion could be substantial, as many vertebrates are facultative scavengers^18,19^. Scavenging can play an important role in trophic webs because it provides more net energy than predation^20^. Animals may also shift from predation to scavenging when a large amount of carrion becomes available^21^.

Although scavengers can rapidly remove some carcasses from roads e.g.^22–25^, few studies have identified which scavengers are responsible for carcass removal (but see^26,27^). As a result, there is currently limited understanding of which scavengers benefit most from this resource. Carrion from vehicle collisions could serve as a food subsidy that increases scavenger populations. Population increases of scavengers may have impacts through increasing human-wildlife conflicts^28,29^ and contributing to declines of imperiled fauna^30,31^. An important step in examining how carrion from vehicle collisions could subsidize scavenger populations is to determine the ecological underpinnings of carrion consumption patterns near roads.

Several factors may influence the degree to which scavengers use carrion resources available on roads. The presence of larger and more dominant predators can influence foraging behavior by prohibiting potential prey species from accessing certain habitats due to predation risk^32,33^. Forested habitats can be less risky for some species because trees provide cover and protection from potential larger predators^34,35^. In contrast, open areas such as those along roads provide little cover and some species may have higher predation risk in these areas^34^. For example, pine martens (*Martes martes*), which climb trees to escape larger predators, avoid clear cuts and scavenge most often in forests where they are more adept at avoiding predation^36,37^. The risk of predation in open areas along roads may discourage scavenging by less dominant species and favor carcass consumption by larger and more dominant carnivores. Conversely, some animals may face reduced predation risk near roads due to predator release^38^.

In addition to minimizing risks, optimal foraging theory predicts that animals should forage to maximize energetic gains, though foraging is often influenced by a suite of environmental and biological factors. Increasing net energy gains can be accomplished in part by concentrating foraging in places with reliable food availability^39,40^. Vultures (Families Cathartidae and Accipitridae, Subfamilies Aegypiinae and Gypaetinae) are obligate scavengers that subsist almost exclusively on carrion^41^. When vultures forage, they use soaring flight to cover large areas while minimizing energetic costs, which is essential for obligate scavenging^41,42^. Because they have a limited ability to shift to other food resources, vultures could be expected to heavily use areas that reliably contain carrion to further reduce the energetic costs associated with foraging.

Vultures alter foraging behavior in accordance with resource availability, as access to food is a primary factor influencing home range ecology of birds^43,44^. Griffon vultures (*Gyps fulvus*) concentrate activities in areas with high densities of hunter kills^45^ and king vultures (*Sarcoramphus papa*) fly along paths used by jaguars (*Panthera onca*) to locate jaguar kills^46^. Griffon vultures also arrive sooner at carcasses placed repeatedly in the same location than at carcasses placed in unpredictable locations, indicating that they more frequently visit areas where they have previously encountered carrion^47^. Food availability is the greatest predictor of Egyptian vulture (*Neophron percnopterus*) space use and individuals made repeated movements of up to 250 km to cattle pens where food was reliable^48^. Reliable presence of carrion along roads could thus cause vultures to travel along roads due to increased foraging opportunities^26^. However, flying over roads could also benefit vultures through rising thermal drafts from the road surface, which minimize the energy required for flight^49^. In Pennsylvania and Maryland, both black vultures (*Coragyps atratus*) and turkey vultures (*Cathartes aura*) foraged mostly in open habitats with roads, but rarely foraged on carcasses along roads^50^, apparently using roads for thermal drafts rather than foraging.

Similarly, use of roads could offer multiple benefits to mammalian scavengers and road use has been documented in many species (e.g.^51–53^). Road clearings may facilitate travel because the vegetation within road clearings typically consists of grasses that are periodically mowed, making movement along roads more energetically efficient than in adjacent habitat. Mammals might select roads for travel because they generally use landscapes in a way that minimizes energy expenditures during movement^54^. Wolves (*Canis lupus*), for example, use seismic lines (narrow linear corridors through forests cleared for energy exploration) because they enable quicker movement, allowing wolves to increase encounter rate of prey^55^. Roads might also have a high abundance of rodents because of the altered vegetation^56,57^, so increased prey availability could further account for use of roads by mammalian scavengers. The multiple benefits provided by roads to vultures and mammals makes it unclear whether they use roads for scavenging opportunities or for other purposes, and exploit carrion as a byproduct of this use.

We explored the influence of roads on vertebrate scavenging by comparing scavenging of rabbit carcasses placed along road verges, along linear features without roads (i.e. power line clearings), and in the forest interior. We hypothesized linear features influence detection and use of carrion by scavengers. We predicted large mammalian predators (i.e. coyotes *Canis latrans*) would use roads most frequently and arrive at them first due to ease of travel and carrion availability from vehicle collisions. We predicted power lines would be used second most frequently by these species because they would provide the same ease of travel as roads, but would provide less carrion. We predicted that forests would be used least often and have the longest arrival times. We further predicted that obligate avian scavengers (i.e. vultures) would exhibit the same patterns as large mammalian predators, with greatest use of roads due to scavenging opportunities and thermals for soaring. For mesomammals (e.g. Virginia opossums *Didelphis virginiana*, gray fox *Urocyon cinereoargenteus*) we predicted greatest use of carrion within forest sites due to reduced exposure to larger predators (i.e. coyotes).

## Results

**Figure 1:**
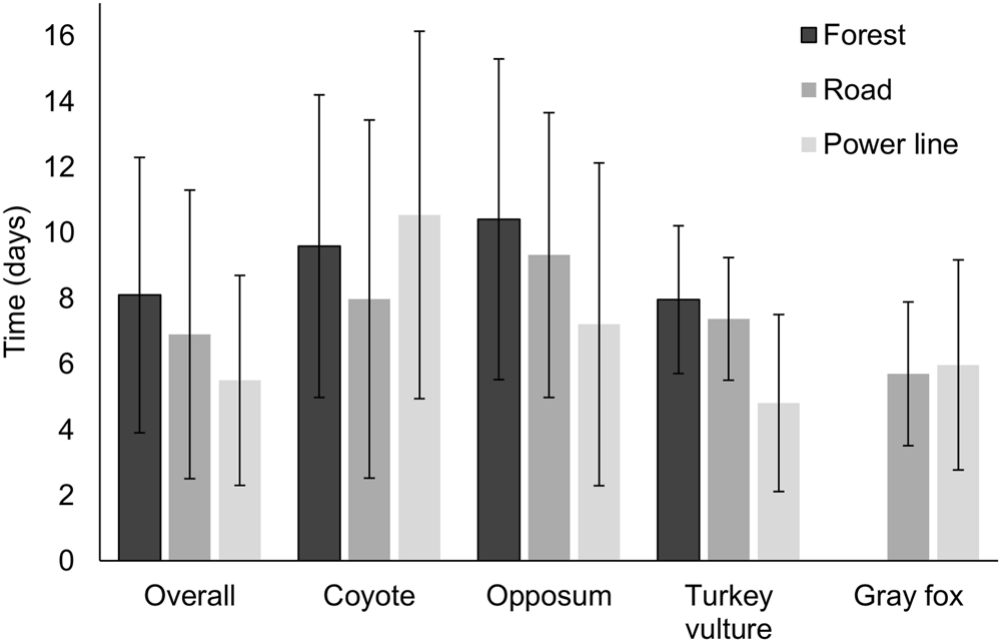
Mean arrival time in days with standard deviations of the first scavenger overall and first visit by various species to rabbit carcasses at forest interior, road, and power line sites. All locations were in pine forests >20 yrs old at the Savannah River Site, Aiken, SC, December 2016-March 2017.

**Figure 2:**
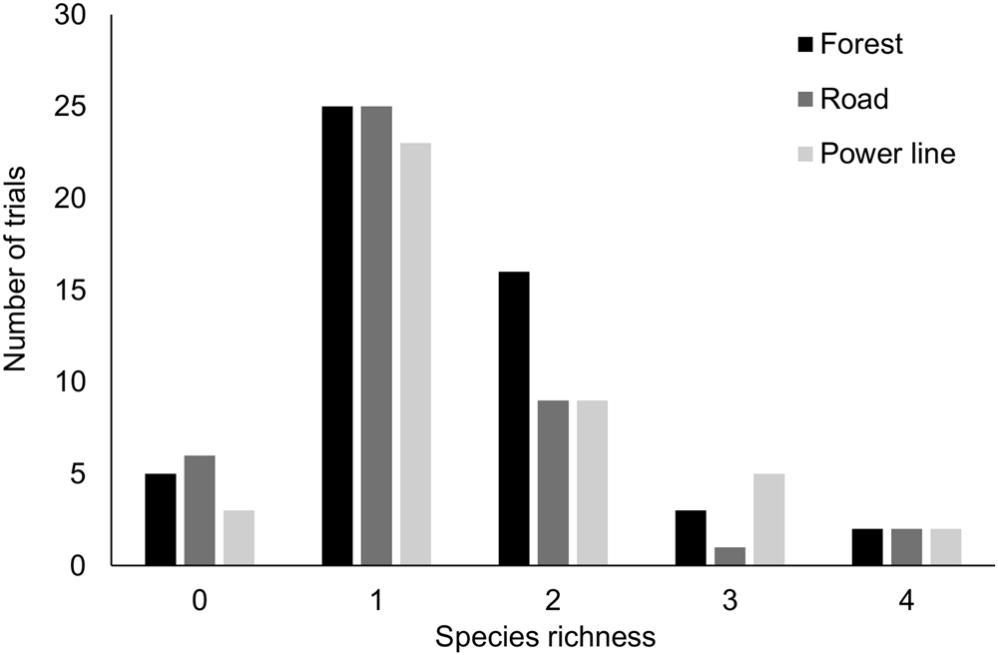
Species richness (i.e. number of species) of scavengers foraging on rabbit carcasses placed in forest interior, road and power line sites. All locations were in pine forests >20 yrs old at the Savannah River Site, Aiken, SC, December 2016-March 2017.

After removing trials due to camera failure, our sample size for analysis consisted of 51 forest, 42 power line, and 43 road trials. Across all treatments we documented 17 species scavenging on carcasses, including 11 species in forest, 12 species in power line, and 11 species in road trials (Table 1). There was no difference in whether a carcass was scavenged across treatments (Table 2); 90% of forest, 92% of power line, and 86% of road trials were scavenged. Mean time to first arrival of a scavenger was shorter at power line sites (5.5 ± 3.2 d) than forest sites (8.1 ± 4.2 d; *β* = −2.60, *P*-value = 0.013), but no other pairwise comparisons were significant (mean road arrival time = 6.9 ± 4.4 d; Fig. 1). Mean species richness was 1.5 ± 0.9 for forest, 1.5 ± 0.9 for power line, and 1.3 ± 0.9 for road trials and there were no significant differences between treatments (forest vs. road *β* = 0.144, *P*-value = 0.698; forest vs. power line *β* = −0.049, *P*-value = 0.956; power line vs. road *β* = 0.193, *P*-value = 0.547; Fig. 2).

**Table 1:** Species documented scavenging rabbit carcasses in pine forests at the Savannah River Site, Aiken SC, December 2016-March 2017.

Species	Forest	Power line	Road	Total
Coyote (*Canis latrans*)	21	18	17	56
Turkey vulture (*Cathartes aura*)	12	17	8	37
Virginia opossum (*Didelphis virginiana*)	21	6	9	36
Gray fox (*Urocyon cinereoargenteus*)	0	4	11	15
Red-shouldered hawk (*Buteo lineatus*)	5	4	1	10
Red-tailed hawk (*Buteo jamaicensis*)	5	4	0	9
Raccoon (*Procyon lotor*)	3	2	3	8
Wild pig (*Sus scrofa*)	1	4	1	6
Bobcat (*Lynx rufus*)	1	1	2	4
Great horned owl (*Bubo virginianus*)	1	1	0	2
American crow (*Corvus brachyrhynchos*)	0	2	0	2
Barred owl (*Strix varia*)	1	0	0	1
Black vulture (*Coragyps atratus*)	0	1	0	1
Southern flying squirrel (*Glaucomys volans*)	1	0	0	1
Striped skunk (*Mephitis mephitis*)	0	0	1	1
Domestic dog (*Canis lupus familiaris*)	0	0	1	1
Red fox (*Vulpes vulpes*)	0	0	1	1
Scavenged	46	39	37	122
Unscavenged	5	3	6	14
Proportion Scavenged	0.90	0.92	0.86	0.90
Mean time to first scavenger arrival (d)	8.1 ± 4.2	5.5 ± 3.2	6.9 ± 4.4	6.9 ± 4.3

Values indicate number of carcasses at which each species was present for each treatment.

**Table 2:** Comparisons of rabbit carcasses scavenged by all vertebrates combined across treatments using a generalized linear model with logit link and time to first scavenger arrival using a generalized linear model with identity link.

Metric	Comparison	Estimate	*p*
Carcasses scavenged	Forest vs road	0.400	0.808
	Forest vs power line	−0.346	0.892
	Road vs power line	−0.746	0.574
Time to first scavenger arrival	Forest vs road	1.214	0.395
	Forest vs power line	2.595	0.013
	Road vs power line	1.382	0.328

Carcasses were placed in pine forests >20 yrs old at the Savannah River Site, Aiken, SC, December 2016-March 2017. A significance level of 0.05 was used for all models.

Four species met our criteria for further analyses: coyote, turkey vulture, Virginia opossum, and gray fox (*Urocyon cinereoargenteus*). There was no difference in coyote presence or arrival times across treatments (Tables 3 and 4). Mean coyote arrival times in days were 9.59 ± 4.61 for forest, 10.54 ± 5.60 for power line, and 7.98 ± 5.46 for roads. Turkey vultures scavenged equally across treatments. Mean turkey vulture arrival times in days were 7.96 ± 2.25 for forest, 4.81 ± 2.70 for power line, and 7.37 ± 1.87 for road trials. Turkey vultures arrived at power line carcasses sooner than those at roads (*β* = −2.56, *P*-value = 0.043) or in the forest (*β* = −3.15, *P*-value = 0.001). Opossums scavenged at forest sites more often than those at power lines (*β* = −1.43, *P*-value = 0.017) but no other comparisons were significant. Mean opossum arrival times in days were 10.41 ± 4.89 for forest, 7.21 ± 4.92 for power line, and 9.32 ± 4.34 for road trials and there was no difference across treatments (Table 4). Gray foxes never scavenged in the forest and there was no difference in presence (*β* = 1.18, *P*-value = 0.118) or arrival time (*β* = 0.20, *P*-value = 0.900) between road and power line treatments. Mean gray fox arrival time in days was 5.97 ± 3.20 for power line and 5.77 ± 2.19 for road trials.

**Table 3:** Presence/absence comparisons of scavenger species at rabbit carcasses analyzed with a generalized linear model with logit link.

Species	Comparison	Coefficient	*p*
Coyote	Forest vs road	0.068	0.986
	Forest vs power line	−0.069	0.985
	Road vs power line	−0.137	0.948
Turkey vulture	Forest vs road	0.504	0.574
	Forest vs power line	−0.586	0.383
	Road vs power line	−1.090	0.076
Opossum	Forest vs road	0.973	0.100
	Forest vs power line	1.435	0.017
	Road vs power line	0.463	0.702
Gray fox*	Road vs power line	1.184	0.118

Carcasses were placed in pine forests >20 yrs old at the 627 Savannah River Site, Aiken, SC, December 2016-March 2017. A significance level of 0.05 was 628 used for all models.

^*^No comparisons with forest because gray foxes did not scavenge in the forest.

**Table 4:** Comparison of arrival times of scavenger species at rabbit carcasses between treatments analyzed using a generalized linear model with identity link.

Species	Comparison	Coefficient	*p*
Coyote	Forest vs road	1.616	0.634
	Forest vs power line	−0.945	0.846
	Road vs power line	−2.560	0.344
Turkey vulture	Forest vs road	0.595	0.851
	Forest vs power line	3.154	0.001
	Road vs power line	2.560	0.043
Opossum	Forest vs road	1.095	0.843
	Forest vs power line	3.200	0.342
	Road vs power line	2.105	0.698
Gray fox*	Road vs power line	−0.200	0.899

Carcasses were placed in pine forests >20 yrs old at the Savannah River Site, Aiken, SC, December 2016-March 2017. A significance level of 0.05 was used for all models.

*Some pairwise comparisons not calculated due to small sample sizes.

For mesomammals pooled, there was no difference in presence across treatments (forest vs. road *β* = 0.12, *P*-value = 0.958; power line vs. road *β* = −0.69, *P*-value = 0.300; power line vs forest *β* = −0.80, *P*-value = 0.167). There was also no difference in arrival times across treatments for mesomammals (forest vs. road *β* = 3.25, *P*-value = 0.053; power line vs. road *β* = −0.48, *P*-value = 0.956; power line vs forest *β* = −3.73, *P*-value = 0.054). There was no difference in whether a carcass was scavenged between the high and medium traffic sites (*β* = −1.609, *P*-value = 0.208) or arrival time of the first scavenger (*β* = −0.083, *P*-value = 0.961). Bootstrapped mean Shannon-Weiner index values and 95% confidence intervals for forest, road, and power line sites, respectively, were 1.738 [1.572, 1.892], 1.801 [1.610, 1.970], and 1.932 [1.761, 2.096].

## Discussion

We did not find support for our prediction that linear features influenced detection of carrion by scavengers. There was no difference in the overall proportion of carcasses scavenged or time to arrival of the first scavenger between the road and other treatments. No species arrived at carcasses near roads sooner than at other treatments. Mesomammals as a group scavenged equally across treatments. Both coyotes and turkey vultures scavenged equally across all treatments, and opossums scavenged equally between roads and forests. Additionally, species diversity did not differ among treatments. Although the scavenger community overall did not show a substantial response to roads or power lines, exclusive consumption by gray foxes of carrion along linear features indicates that species differ in their use of these features for scavenging compared to forests. We suggest differences in scavenging by species across treatments were influenced by resource distribution, sensitivity to vehicle traffic, and habitat and diet flexibility.

In contrast to our expectations, vultures did not scavenge most frequently along roads. Vultures are thought to make considerable use of carrion on roads at the Savannah River Site (SRS)^44,58^, but would only be expected to focus activities on roads if there was a greater chance of encountering food in these locations than in surrounding habitat^40^. In Africa, where food was evenly distributed across the landscape, Egyptian vultures exhibited a Brownian movement strategy while foraging^59^. This seemingly random movement resulted from the uniform distribution of carrion because concentrating activities in a particular area did not increase the probability of foraging success. At our site, carrion may be similarly distributed evenly across the site such that foraging success is not increased by concentrating activities along roads.

In addition to resource distribution, resource abundance may have also influenced vulture foraging behavior. Turkey vulture home ranges have decreased substantially at SRS over the past decade^58,60^, which may be due to an increase in carrion availability provided by a growing wild pig population^61^. Pig carcasses left in the field from management activities could create a large supply of carrion on which vultures may be feeding (see Methods). However, carcasses are distributed across the site in an unpredictable pattern, so carrion should still be more reliably available along roads than in the forest. Additionally, there is a landfill at SRS and vultures in many locations regularly scavenge at landfills e.g.^62,63^, further increasing food supply. The abundance of food for vultures at SRS may negate any benefits of concentrating foraging activities in a particular area and may partially account for the lack of increased scavenging along roads.

Traffic on roads could impact scavenging by vultures since they are diurnal and their highest activity levels coincide with times of heavy traffic at SRS. Some birds, including turkey vultures, may not be able to avoid rapidly approaching vehicles, putting them at risk of collision^64,65^. Both cinereous vultures (*Aegypius monachus*) and Griffon vultures decrease presence near roads as traffic volume increases^66^. Andean condors (*Vultur gryphus*) spent more time vigilant when foraging near roads, indicating that they perceived roadside areas to be of higher risk than areas further away from roads^67^. Vultures arrived at power line carcasses quicker than at roads, which may be the result of decreased vigilance due to lack of vehicle traffic. However, the quicker arrival times may have also resulted from our spatial arrangement of carcasses. We did not detect a difference in scavenging across traffic levels, which may have occurred because there was not enough difference in traffic to impact scavenger behavior. Vultures scavenged equally across road and forest sites, but the risk of vehicle collision may account for lack of increased scavenging near roads.

Opossums overall did not scavenge less frequently in open areas than in the forest. Although they scavenged less frequently along power lines than in the forest, the lack of difference in scavenging between roads and the forest suggest that avoidance of open areas was not a primary driver of scavenging behavior. In Indiana, density of adjoining roads was a significant predictor of opossum density^68^ and opossums selected habitat in close proximity to roads throughout much of the year^53^. Although coyotes consume opossums (e.g.^69–71^), they may not pose enough of a risk to substantially alter habitat use of opossums at SRS. As generalists, opossums often thrive in anthropogenically modified habitats and our results suggest they have the ability to exploit carrion in both forests and along roads.

Coyotes are also generalists and scavenging by coyotes was ubiquitous across all three treatments with no differences in arrival times. This may have been influenced by the habitat present at our sites, as mature pine stands generally have little undergrowth that would impede movement by coyotes. Additionally, most pine stands on the SRS are routinely subjected to prescribed burns, which would have further reduced the understory vegetation. As a result, movement along roads may have only been marginally more energetically efficient than moving through the forest, resulting in equal use of carcasses across treatments. In habitats with denser understory vegetation, movement might have been substantially more energetically costly, and placing carcasses in such habitats could potentially result in decreased carrion consumption compared to roads.

Gray foxes were the only species that scavenged more frequently along linear features than in the forest and there was no difference in arrival time between power line and road treatments. Use of corridors may have been driven by prey availability, as small mammal abundance is an important determinant of gray fox habitat selection^72^ and road verges may have increased rodent densities^57,73,74^. The amount of scavenging by gray foxes was more than twice that reported by other studies at SRS due to our focus on linear features. Turner, *et al*.^75^ reported scavenging by gray foxes at 4.3% of rabbit carcasses during winter, whereas we documented gray foxes scavenging at 9.6% of carcasses. The former study examined scavenging in forest interior sites and clear cuts and did not examine use of carrion along linear features. Had we examined forest interior sites exclusively, gray foxes may have never been documented, even though they were the fourth most common scavenger overall. Although species diversity was similar across treatments, the composition of those species was thus different between the linear features and forest sites due to lack of gray fox scavenging in the latter.

At SRS, space use of gray foxes may be influenced by avoidance of coyotes, as gray foxes have been shown to select core home range areas that do not have high concentrations of coyotes^76^. Furthermore, the mature pine stands in which we placed carcasses are the second most selected habitat by coyotes at SRS^77^. As a result, any potential protection from predation afforded to gray foxes by vegetative cover in mature pine stands may be offset by increased use of the habitat by potential predators, leading to minimal use of carrion by gray foxes in these habitats. Similarly, gray foxes in Georgia selected roads, but used mature pine stands at random^78^. Roads and power lines may have provided efficient travel corridors for gray foxes, allowing them to move through areas of high use by coyotes while minimizing predation risk.

Scavenging patterns were likely influenced by the time of the year in which the study took place, as mammals often scavenge more extensively during cooler seasons e.g.^37,75^. Increase in decomposition rate during warm weather often makes carcasses unpalatable for mammals before they can be detected^18^. Coyotes and opossums scavenged frequently in our study, but they seldom scavenge rabbit carcasses during summer at SRS^75,79^. Decomposition of carcasses during warmer temperatures provides olfactory cues used by turkey vultures to detect carrion and slower decomposition rate could diminish their ability to find carcasses e.g.^80,81^. Because black vultures frequently use presence of turkey vultures at carcasses for detection, season may also account for lack of scavenging by black vultures^75^. Consequently, the patterns of carcass consumption we documented during winter may be much different than what occurs during warmer seasons. Additionally, we did not have data on animal abundance across habitats in our study. Comparing presence of scavenger species at carcasses to their abundance in respective habitats may have produced different conclusions and should be considered in future studies.

Our results indicate that anthropogenic linear features such as roads can result in differences in the scavenger community across small spatial scales (i.e. 500 m apart) within the same habitat, because gray foxes did not scavenge in forests. If roadside habitat offers benefits to a species such as reduced predation risk compared to adjacent habitat, the presence of a road may lead to occurrence of the species in locations it may otherwise avoid. Interactions between species may influence carcass use along linear features and consideration of such interactions is necessary to fully understand the dynamics of scavenger communities.

The similarly high consumption of carrion across all treatments suggests that carcasses are consumed by vertebrates at forested habitats at this site with minimal regard to the presence of linear features. Although some species may benefit from increased carrion availability from wildlife-vehicle collisions, they may also be at risk of being killed by a vehicle while scavenging along roads. Research in urban areas has shown similar high consumption of carcasses on roads by vertebrates^82^. Carrion along roads can be consumed by invasive species and facilitate their range expansions by supplying food^83^. The potential for roads to be an attractant due to increased foraging opportunities could be an important yet little-studied mechanism by which human activities impact vertebrate scavengers.

## Methods

### Study site

We conducted this study at the Savannah River Site (SRS), a property owned by the US Department of Energy that encompasses 78,000 ha across Aiken, Allendale, and Barnwell Counties in South Carolina, USA (33°19′N, 81°42′W). SRS is dominated by loblolly pine forests (*Pinus taeda*), longleaf pine forests (*Pinus palustris*), and bottomland hardwoods (e.g. *Nyssa* spp., *Quercus* spp.)^84^. Since 1951, much of SRS has been managed for timber harvest and stands are harvested on a rotating basis^84^. There is a growing population of invasive wild pigs at SRS. The estimated density of adult wild pigs at SRS is 2.6 pigs/km^2^, equivalent to approximately 2000 adults^85^. The inclusion of yearlings and piglets makes the total estimated number of individuals at SRS 4000-6000^85^. Management of the wild pig population at SRS involves killing a substantial number of individuals and carcasses are often left in the field.

There are 225 km of primary (i.e. paved) roads and 2250 km of secondary roads on the SRS^86^. We conducted this study December 2016–March 2017; mean monthly temperature ranged from 10.9–14.4 °C and mean daily precipitation was 3.9 mm^87^. We chose to carry out the study during winter because scavenging by mammals at SRS is infrequent during summer^75,79^, which would have made it difficult to attain a sample size large enough to test our hypotheses.

### Study design

**Figure 3:**
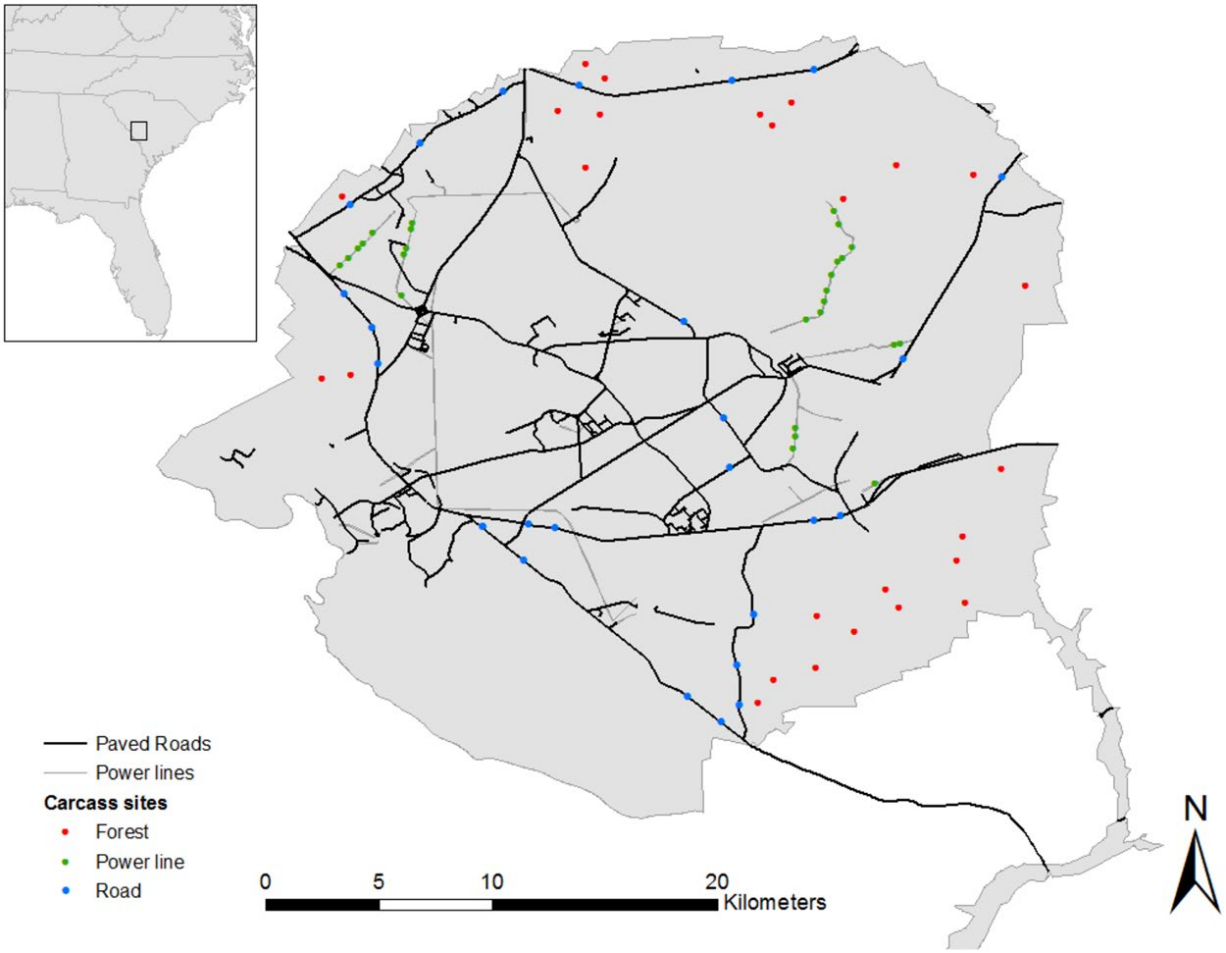
Map of Savannah River Site with locations of rabbit carcass placement across three treatments: roads, power lines, and forests. All carcasses were placed in pine forests >20 yrs old and were separated by at least 500 m. Box in inset map shows location of the study site.

We selected 78 sites in pine (*Pinus* spp.) stands that were greater than 20 years old (Fig. 3), which we divided evenly among three treatments: power line clearings, roads, and forest. We chose power line clearings as linear features because they are common on the site and similar in width and vegetation structure to most clearings for roads, but have less carrion availability. Although carrion can be present on power lines due to avian collisions, more than nine times as many birds die on roads compared to power lines annually in the United States^88^. Furthermore, mammals are unlikely to die along power line clearings because they cannot come into contact with power lines. The single-pole power lines along which we placed carcasses are unlikely to be sites of substantial avian mortality. Thus we believe our assumption of substantially less carrion availability on power lines compared to roads at this site to be reasonable.

All roads were two-lane paved roads and all road and power line sites contained forested habitat on either side. Forest interior sites were located at least 500 m from the forest edge. We separated all sites by at least 500 m and every site was at least 500 m from the next nearest road or power line. Our number of sites and distance between sites was based on the maximum number of locations available meeting these restrictions concerning habitat and distance to proximate linear features. It was not possible to choose road sites with equal traffic levels and maintain our site selection criteria. Of the road sites, 14 were on public access roads that were heavily trafficked, 9 were not publicly accessible but were frequently traveled by personnel on site, and 3 were not publicly accessible and experienced little traffic. We designated these sites high, medium, and low traffic sites, respectively. Number of vehicles per hour at high traffic sites reaches the thousands during times of peak activity^89^. We performed four consecutive rounds of 39 trials that lasted three weeks each for 156 total trials. For the first round, we selected 13 sites from each of the three treatments and used the remaining sites in the second round. Proximate sites were not used at the same time so that there was a minimum of 1 km between sites used concurrently. Alternate sites were used in consecutive rounds such that each site was used twice with about three weeks between reuse of sites.

At each site we placed a dark colored rabbit (*Sylvilagus* spp.) carcass weighing ~1300 g obtained from a commercial supplier (RodentPro, Inglefield, IN, USA) and thawed indoors to ambient temperature. We used a cable lock to attach a motion-activated infrared camera (Bushnell Trophy Cam HD Aggressor; Bushnell Corp., Overland Park, KS, USA) to a tree ~3 m from carcasses to record the presence of scavengers. At road and power line sites, there was no tree cover over carcasses so they would be visible from overhead. At road sites, carcasses were placed along verges ~3 m from the road pavement to reduce the risk of animals being struck by a vehicle while scavenging. Cameras took three pictures when motion-activated, with a 1-minute delay between activations. To prevent scavengers from carrying carcasses beyond the field of view, we affixed a non-relaxing cable snare to each carcass and staked it to the ground with a 46-cm steel rebar stake.

We used images from the cameras to identify the scavenger species present at each carcass. We compared whether each carcass was scavenged (i.e. presence/absence of any scavenger) across the three treatments using a generalized linear model with binomial distribution and logit link using R version 3.2.3^90^. We compared the time between carcass placement and first arrival of any scavenger species between treatments using a generalized linear model with Gaussian distribution and identity link. We also calculated species richness (i.e. cumulative number of species present) at each carcass and compared across treatments using a generalized linear model with Poisson distribution and log link. This was done to investigate differences in scavenger community structure across treatments. We also compared presence/absence and arrival time of all mesomammals pooled together. This set of species included Virginia opossum, gray fox, raccoon (*Procyon lotor*), striped skunk (*Mephitis mephitis*), bobcat (*Lynx rufus*) and red fox (*Vulpes vulpes*). If a species was present at 15 or more carcasses in total, we similarly compared the presence/absence of the species and arrival time of the species across treatments. To examine whether the amount of traffic influenced scavenging behavior, we compared presence/absence of scavenging and time to arrival of the first scavenger as described above between high and medium traffic road sites (sample size of low traffic roads was too small for analysis). A significance level of 0.05 was used for all models. When three-way comparisons were significant, we used a Tukey’s HSD test for pairwise comparisons.

We compared species diversity between treatments using the Shannon-Weiner index to further examine scavenger community structure across treatments. We pooled all trials from each treatment using the number of species that were present at each carcass (Table 1). This was done because animals frequently attempted to remove carcasses from the snares and number of visits recorded at a carcasses would have depended on the ability to dislocate the carcass and was not an ecologically relevant metric. Thus there was a single species diversity value for each treatment and we used R to bootstrap these values with 1000 replications to obtain 95% confidence intervals. The datasets generated during this study are available in the Dryad repository (10.5061/dryad.7kj575k).
